# IΚΚε cooperates with either MEK or non-canonical NF-kB driving growth of triple-negative breast cancer cells in different contexts

**DOI:** 10.1186/s12885-018-4507-2

**Published:** 2018-05-25

**Authors:** Carrie D. House, Valentina Grajales, Michelle Ozaki, Elizabeth Jordan, Helmae Wubneh, Danielle C. Kimble, Jana M. James, Marianne K. Kim, Christina M. Annunziata

**Affiliations:** Women’s Malignancies Branch, National Cancer Institute, Bethesda, MD USA

**Keywords:** NF-kappaB, Triple negative breast cancer, IKK-epsilon, Non-canonical signaling, Anoikis

## Abstract

**Background:**

Metastatic breast cancer carries a poor prognosis despite the success of newly targeted therapies. Treatment options remain especially limited for the subtype of triple negative breast cancer (TNBC). Several signaling pathways, including NF-κB, are altered in TNBC, and the complexity of this disease implies multi-faceted pathway interactions. Given that IKKε behaves as an oncogene in breast cancer, we hypothesized that IKKε regulates NF-κB signaling to control diverse oncogenic functions in TNBC.

**Methods:**

Vector expression and RNA interference were used to investigate the functional role of IKKε in triple-negative breast cancer cells. Viability, protein expression, NF-κB binding activity, invasion, anoikis, and spheroid formation were examined in cells expressing high or low levels of IKKε, in conjunction with p52 RNA interference or MEK inhibition.

**Results:**

This study found that non-canonical NF-κB p52 levels are inversely proportional to ΙΚΚε, and growth of TNBC cells in anchorage supportive, high-attachment conditions requires IKKε and activated MEK. Growth of these cells in anchorage resistant conditions requires IKKε and activated MEK or p52. In this model, IKKε and MEK cooperate to support overall viability whereas the p52 transcription factor is only required for viability in low attachment conditions, underscoring the contrasting roles of these proteins.

**Conclusions:**

This study illustrates the diverse functions of IKKε in TNBC and highlights the adaptability of NF-κB signaling in maintaining cancer cell survival under different growth conditions. A better understanding of the diversity of NF-κB signaling may ultimately improve the development of novel therapeutic regimens for TNBC.

**Electronic supplementary material:**

The online version of this article (10.1186/s12885-018-4507-2) contains supplementary material, which is available to authorized users.

## Background

Breast cancer results in approximately 40,000 deaths per year in the United States [1]. Despite new therapies designed to target different subtypes of breast cancer, there remains a poor prognosis for metastatic disease. A further understanding of the molecular mechanisms required for tumor cell survival during the process of metastasis and relapse will aid in the development of more targeted therapies for breast cancer, especially in the case of the triple-negative breast cancer (TNBC) subtype for which treatment options are limited.

TNBC is a heterogeneous disease characterized by an absence of well-defined targets and a poor five-year survival rate for metastatic disease. Gene expression and ontology studies have further characterized TNBC into as many as six molecular subtypes that include basal-like and non-basal like tumors [2, 3]. Recognition of individual subtypes in patients will likely lead to better therapeutic strategies; the complexity associated with TNBC, however, implies an increased level of pathway cross-talk and compensatory mechanisms [4]. Signaling pathways altered in TNBC progression include p53, PI3K, MEK, and NF-κB, among others [4–10] with new clinical trials underway using novel combinations of different pathway inhibitors [4].

NF-κB is a signaling pathway important for controlling immune response, stress response, cell survival, proliferation, differentiation, and apoptosis [11–15]. Activation of NF-κB is mediated by the IκB kinases IKKα, IKKβ, and ΙΚΚε, resulting in nuclear localization of NF-κB transcription factors (c-Rel, RelA, RelB, p105/50 and p100/52) and subsequent transcriptional activation [16]. There are both canonical and non-canonical pathways operating within the NF-κB network, allowing tight regulation of various biological functions [17]. Activation of NF-κB can occur through multiple stimuli, and this pathway interacts with other prominent signaling pathways, although the molecular mechanisms contributing to cancer progression remain unclear [16, 18–21]. Our laboratory has previously characterized NF-κB activation downstream of IKKβ and IKKε in ovarian cancer, and several studies support that NF-κΒ is an important contributor to cancer progression and chemoresistance [17, 22–24]. Expression analysis of TNBC tissue with adjacent normal breast tissue suggests that NF-κB is a key regulator of the molecular TNBC phenotype [5].

The kinase ΙΚΚε (encoded by *IKBKE* gene) has been shown to be an oncogene in breast [20, 25, 26] and ovarian [24] cancers. Silencing of *IKBKE* reduced proliferation, clonogenicity, migration and invasion of breast cancer cells [20, 27]. ΙΚΚε, in cooperation with MEK, can function as a transforming kinase in human mammary epithelial cells [20]. Most studies have focused on ΙΚΚε function in the luminal subtype, whereas the role of this kinase in the more aggressive basal subtype has only recently been explored. In that setting, ΙΚΚε in combination with Jak/Stat signaling may promote cytokine activation that induces tumorigenesis in an immune-activated subtype of TNBC. Although ΙΚΚε is known to phosphorylate one of two acceptor sites of IκBα, its role in NF-κB activation remains unclear. Given the broad activity of NF-κB, our work presented here seeks to clarify whether this kinase supports canonical or non-canonical signaling and, furthermore, what oncogenic features depend on this signaling circuit.

## Methods

### Cell lines and culture conditions

Breast cancer cell lines MDA MB 231 (cat. No. HTB-26) [claudin-low TNBC], MDA MB 453 (cat. No. HTB-131) [HER2 (ER-,PR-, HER2+)], MDA MB 468 (cat. No. HTB-132) [basal TNBC], HCC-38 (CRL-2314) [claudin-low TNBC], BT-549 (cat. No. HTB-122) [basal TNBC], and BT-474 (cat. No. HTB-20) [luminal B (ER-, PR+,HER2+] were purchased from American Type Culture Collection (ATCC, Manassas, VA). Unless otherwise noted, all breast cancer cell lines were cultured in RPMI 1640 (Gibco, Thermo Fisher, Grand Island, NY) containing 10% FBS (Gemini, West Sacramento, CA) and 1% penicillin/streptomycin (Gibco, Thermo Fisher, Grand Island, NY) and maintained at 37°C in a 5% CO_2_ atmosphere.

### Expression and shRNA constructs

pBabeNeo (plasmid #1767) and pBabe-Neo-Flag-IKBKE (plasmid #15265) were purchased from Addgene. Transduced cells were cultured in the presence of 200μg/ml neomycin for 7 days. Use of *IKBKE* short hairpin (shRNA) constructs has been previously described [24]. Two rounds of viral supernatants were applied to breast cancer cell lines over the course of 48 h, followed by incubation with growth medium for 24 h and selection with 2 μg/mL puromycin for 7 days. Selected transduced cells were used for all assays. Sequences of shRNA constructs: non-targeting control (shNeg):

- forward *GATCCC*CTCTCAACCCTTTAAATCTGATTCAAGAGATCAGATTTAAAGGGTTGAGAG*TTTTT,* reverse *AGCTAAAAACTCTCAACCCTTTAAATCTGATCTCTTGAATCAGATTTAAAGGGTTGAGAGGG.*

shΙΚΚε 1:

- forward *GATCCCGAGAAGTTCGTCTCGGTCTATTTCAAGAGAATAGACCGAGACGAACTTCTCTTTTT,*
- reverse *AGCTAAAAAGAGAAGTTCGTCTCGGTCTATTCTCTTGAAATAGACCGAGACGAACTTCTCGG.*

shΙΚΚε 2:

- forward GATCCCGAGAGCCTCCTGTTCTTTCTATTCAAGAGATAGAAAGAACAGGAGGCTCTCTTTTT.
- reverse *AGCTAAAAAGAGAGCCTCCTGTTCTTTCTATCTCTTGAATAGAAAGAACAGGAGGCTCTCGG.*

### siRNA transfections

Cells were cultured for 24 h to 50% confluence before transfection with Dharmacon On-Target*plus* SMARTpool short interfering (siRNA) duplexes (NF-kB2, cat. No. L-003918-00; non-targeting control, cat. No. D-001810-10; IKBKE, cat. No. L-003723-00) according to manufacturer’s instructions (GE Dharmacon, Lafayette, CO). Briefly, cells were transfected with Dharmafect 1 transfection reagent (GE Dharmacon, Lafayette, CO) and individual siRNAs at a final concentration of 1% *v*/v and 25 nM, respectively. Cells were maintained in the presence of transfection reagent under normal culture conditions for 24 h before being used in assays.

### RNA extraction and quantitative real-time PCR (qRT-PCR)

Total RNA was extracted using the RNeasy Mini Kit (Qiagen) per manufacturer’s instructions and treated with DNAse. Final RNA concentration was determined using a NanoDrop spectrophotometer. RNA was reverse transcribed using Taqman reagents (Applied Biosystems) and gene expression was measured using Taqman probes on a ViiA7 Real-time PCR machine (Applied Biosystems). *GAPDH* was used as a control and quantitation of gene expression was accomplished using comparative threshold cycle ΔΔC_T_. Primers were purchased from Applied Biosystems (p52 cat. No. Hs01028901_g1, CXCL1 cat. No. Hs00236937_m1, CD44 cat. No. Hs01075861_m1, and GAPDH cat number: 4325792.

### Western blot

Whole cell protein was extracted from breast cancer cell lines using standard methods with NP-40 lysis buffer. Protein concentrations were determined using BCA Protein Assay Kit (Pierce, Thermo Scientific, Rockford, IL). SDS-PAGE was performed using the NuPage system (Invitrogen) and Luminata HRP Chemiluminescent Detection Reagents (Millipore, Temecula, CA). Antibodies were purchased from Sigma (IKKε, cat. No. I4907),

Abcam (IKKβ, cat. No. ab32135), Millipore (GAPDH, cat. No. MAB374; p100/52 cat. No. 05–361), Santa Cruz (p65, cat. No. sc-372), and Cell Signaling (IKKα, cat. No. 2682; pERK1/2, cat. No. 4377; Erk1/2, cat. No. 9102; phospho-p-65 (Ser536), cat. No. 3033).

### Chromatin immunoprecipitation-qPCR (ChIP-qPCR) assay

The SimpleChIP Enzymatic Chromatin IP Kit (magnetic beads) was purchased from Cell Signaling Technology (Danvers, MA). Assays were performed according to the manufacturer’s instructions. The antibody for p52 was purchased from Santa Cruz Biotechnology (cat. No. sc-7386 X). Promo was used to evaluate DNA sequences for transcription factor binding sites (http://alggen.lsi.upc.es/cgi-bin/promo_v3/promo/promoinit.cgi?dirDB=TF_8.3). The first 5000 bases upstream of the transcription start site were screened for binding motifs that correspond to NF-κB consensus binding sequence. The quantification of transcription factor binding to target genes was calculated by measuring the ratio of ChIP-to-Input and normal rabbit IgG antibody served as a negative control. Primer sequences for NF-kB binding sites on *CXCL1* promoter: − 2.5 kb site: forward *GATTTCCAGGCTCAAGGATGTA*, reverse *TCATTCAGTCTTCCAAACAAGC*; − 02. Kb site: forward *ATCCCAGAGTCTCAGAGTCCAC*, reverse *AAATTCCCGGAGTTCCAGAT.*

### Co-immunoprecipitation assay

Immunoprecipitation was performed on MDA MB 468 cells using the Abcam immunoprecipitation kit (cat. No. ab206996), according to manufacturer’s instructions. Briefly, non-denaturing lysis buffer was used to collect 300 μg of cell lysate was incubated overnight with 3 μg/ml of either control rabbit IgG (Santa Cruz, cat. No. sc-2027) or IKKε rabbit polyclonal antibody (Abcam, cat. No. ab7891). Antibody bound proteins were captured using protein A/G sepharose beads, eluted, and analyzed via western blot. Antibodies used for western blot detection were purchased from Sigma (IKKε, cat. No. I4907), Santa Cruz (IKKα, cat. No. sc-7606 and NIK cat. No. sc-8417).

### Viability assay

MEK inhibitor, AZD6244, and a non-selective inhibitor of Ser/Thr kinases, BX795, that inhibits IKKε, among others were purchased from Selleck Chemicals (Houston, TX). The IKKβ inhibitor IKK-2 inhibitor IV from Calbiochem (San Diego, CA). The breast cancer cell growth was assessed using XTT as described [28]. Cells were seeded in 96-well plates at a density of 2000 cells/50 μl/well. Plates were incubated for up to 9 days with medium and/or drug replenished every 3–4 days. Growth was assessed by incubating cultures with XTT for 3 h and absorbance read in a Tecan plate reader (Research Triangle Park, NC). Cell density in experimental wells was expressed as percent control. Experiments included triplicate samples and were repeated at least three times. IC50 values were calculated using CalcuSyn software (Paramus, NJ) and compared with publicly available database (www.cancerrxgene.org).

### NF-κB immunoassay

Nuclear lysates were extracted using a Nuclear Extraction Kit according to manufacturer’s instructions (Active Motif, Carlsbad, CA). Protein concentrations were determined using BCA Protein Assay Kit (Pierce, Thermo Scientific, Rockford, IL). NF-κB transcription factor binding was assessed using the TransAM NFκB Family ELISA Kit according to manufacturer’s instructions (Active Motif, Carlsbad, CA). 10 μg of nuclear extracts/20 μl/well were analyzed in triplicate and repeated at least three times.

### Invasion assay

Invasion potential of breast cancer cells was assessed using Cultrex 96-well BME Cell Invasion Assays (Trevigen, Gaithersburg, MD) according to manufacturer’s specifications. Briefly, 5 × 10^4^ cells suspended in serum-free RPMI were plated in BME coated chambers, and allowed to migrate for 48 h using RPMI containing 10% FBS as a chemoattractant. Cells that migrated through BME chambers were stained with calcein, solubilized, and numbers assessed by measuring fluorescence in a Tecan fluorimeter (Tecan, Research Triangle Park, NC). Migrated cell numbers in triplicate samples were reported as percent control.

### Anoikis assay

Anchorage-independent, low-attachment (LA), growth was evaluated using the CytoSelect 96-Well Anoikis Assay according to manufacturer’s protocol (Cell Biolabs, Inc., San Diego, CA). Briefly, 1 × 10^4^ cells/100 μl/well were plated in quadruplicate on a 96-well anchorage resistant plate and in a companion standard culture plate with cell high attachment capability (HA) plate and cultured for 48 h. Anoikis was assessed by dual staining with calcein AM and ethidium homodimer followed by measurement of fluorescence in a Tecan fluorimeter (Tecan, Research Triangle Park, NC). Experiments were repeated at least three times. Fluorescence intensity was expressed relative to control treatment.

### Spheroid formation assay

To generate breast cancer spheroids, 500 cells/well were cultured in serum free media containing EGF (20 ng/ml) and FGF (10 ng/ml) (Sigma-Aldrich, St. Louis, MO) in ultra-low attachment 96 well plates (Corning, Corning, NY). After 96 h, spheroids were imaged using AxioVision Rel. 4.8 through an Axio Observer A1 Inverted Microscope (Zeiss) and analyzed using ImageJ Software 1.48v [29]. Spheroids with diameter ≥ 50 μm were quantified. Spheroid efficiency was calculated using the formula (spheroids ≥50 μm/ cells per well).

### Statistical analysis

All statistical analysis was performed using Prism (GraphPad) using data acquired from at least three biological replicates. *P* < 0.05 was considered statistically significant. *P* values were calculated as described in figure legends. Error bars represent standard error of the mean.

## Results

**Fig. 1:**
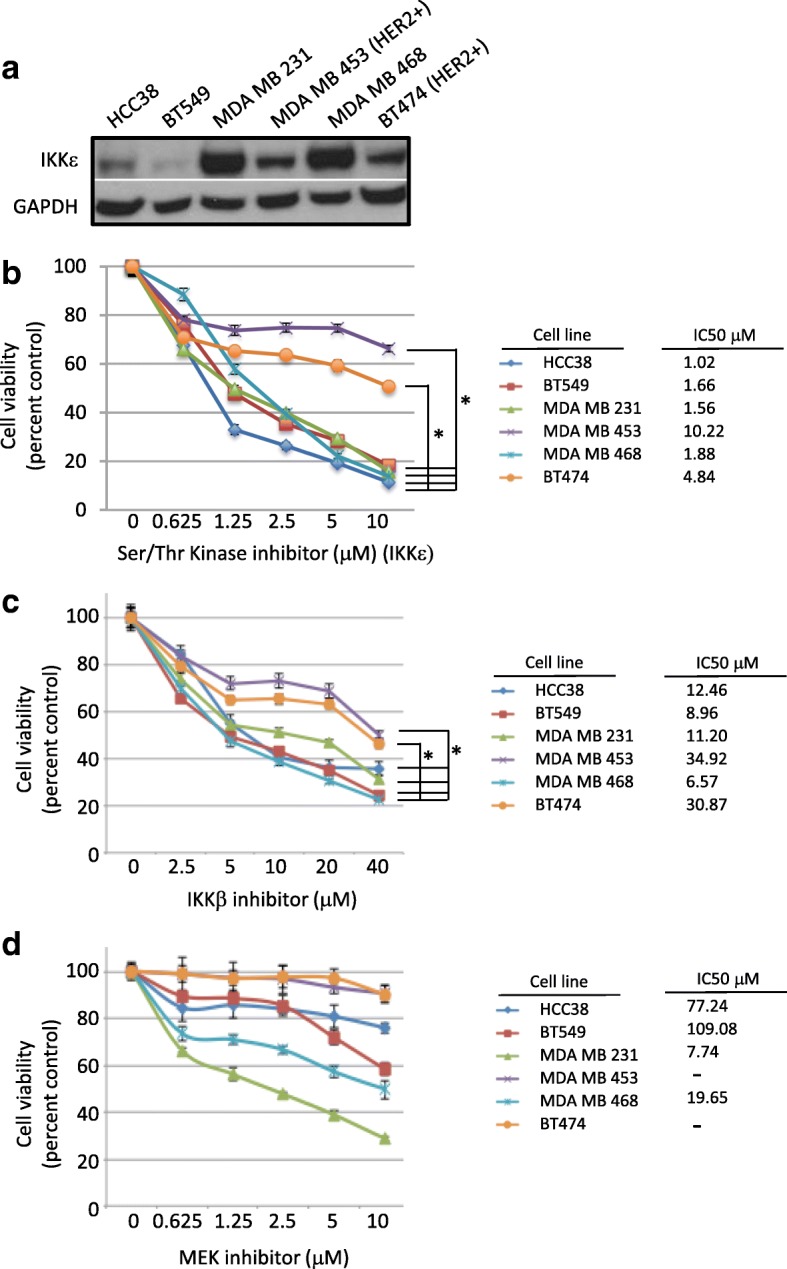
IKKe expression is variable and correlates with sensitivity to MEK inhibition in TNBC subtype. **a**) 30 mg of protein was analyzed in whole cell lysates of breast cancer cells grown to 70% confluence. **b**-**d**) 2,000 cells per well were seeded into 96 well plates and allowed to adhere overnight. Inhibitors were added at indicated concentrations and viability assessed via XTT after 72 hours. * IC50 values significantly different between cell lines as indicated, *P* < 0.05, one-way ANOVA, comparing each cell line individually. Due to the extreme resistance of cell lines MDA MB 453 and BT474, IC50 values could not be reliably calculated, and statistics are therefore not presented

The protein levels of ΙΚΚε in breast cancer cell lines of different molecular subtypes were surveyed. Consistent with previous reports [20], ΙΚΚε expression was variable and independent of basal or luminal status (Fig. 1a). Since ΙΚΚε has been shown to activate the NF-κB pathway and cooperate with MEK to induce transformation in breast cells, the sensitivity of the breast cancer cells to inhibiting ΙΚΚε, IKKβ or MEK was assessed. The relative sensitivities of the breast cancer cells to the indicated inhibitors, based on their calculated IC50 values, were similar to what has been previously reported in the literature (Additional file 1: Table S1). TNBC cells were comparably sensitive to both ΙΚΚε and IKKβ inhibition suggesting dependence on NF-κB classical signaling, whereas the HER2+ cells were more resistant to both inhibitors (*p* < 0.05, Fig. 1b-c). HER2-postitive cells were most resistant to IKKε inhibition, and sensitivity to classical NF-κB pathway inhibition was independent of ΙΚΚε expression level. HER2+ cells were also most resistant to MEK inhibition whereas TNBC cells with the highest ΙΚΚε expression (MDA MB 468 and MDA MB 231) were most sensitive (*p* < 0.05, Fig. 1d). These data corroborate findings by others showing that ΙΚΚε cooperates with MEK to maintain viability.

**Fig. 2:**
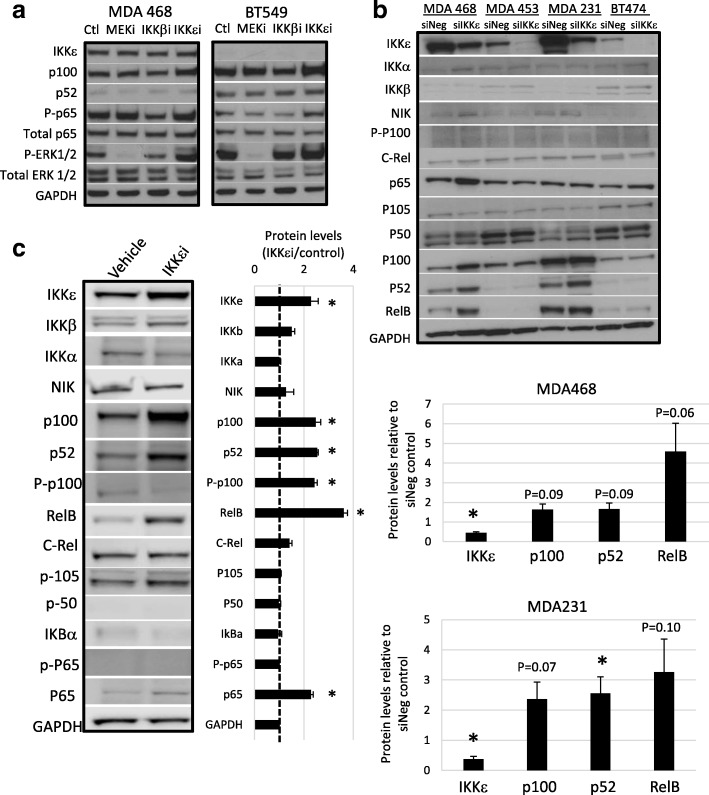
IKKε supports viability and MEK activation. **a**) 30 μg of protein was analyzed in whole cell lysates of breast cancer cells grown to 70% confluence. Cells were treated with vehicle control (Ctl), 2 μM MEK inhibitor (MEKi), 2 μM IKKβ inhibitor (IKKβi), or 2 μM BX795 for inhibition of IKKε (IKKεi) for 6 h before lysate collection. **b**) 30 μg of protein was analyzed in whole cell lysates of breast cancer cells grown to 70% confluence after siRNA mediated knockdown of *IKBKE* (siIKKε). Quantification of three independent replicates reveals increased non-canonical NF-κB proteins with *IKBKE* knockdown. **c**) 30 μg of protein was analyzed in whole cell lysates of MDA MB 468 cells after 1 h exposure to 2 μM BX795 for IKKε inhibition. Quantification of three independent replicates confirms pharmacological inhibition of IKKε activity significantly increased non-canonical NF-κB protein levels. *significantly different from corresponding vehicle control *P* < 0.05, unpaired T-test (**b**-**c**)

A western blot was performed on the TNBC cells MDA MB 468 and BT549, which endogenously express high and low ΙΚΚε, respectively, after 6-h treatment with the inhibitors. The activity of the IKKβ and MEK inhibitors was confirmed through western blot analysis of p65 phosphorylation at serine 536 and phosphorylated ERK1/2 (Fig. 2a and Additional file 2: Figure S1a). Interestingly, IKKβ inhibition reduced phosphorylated ERK1/2 levels only in the presence of ΙΚΚε, suggesting that MEK activation is, at least partially, regulated by canonical NF-κB signaling involving ΙΚΚε. We verified the direct effect of IKKβ inhibition on reduced phosphorylated ERK1/2 at a 30-min time point (Additional file 2: Figure S1b) Inhibition of ΙΚΚε led to an increase in p100/52, p65, and phosphorylated ERK 1/2. Increased protein levels seen with pharmacological ΙΚΚε inhibition were confirmed using an siRNA against *IKBKE* (siIKKε) in the panel of breast cancer cells that express high endogenous IKKε (Fig. 2b). Indeed, there was an increase in p100/52 and RelB with *IKBKE* knockdown that approaches significance. This phenomenon was only apparent in the TNBC cells as non-canonical NF-kB signaling proteins are not highly expressed in the HER2+ cells. The MDA 468 cells were then treated with BX795 to inhibit IKKε for 1 h, a shorter time point, and all NF-κB protein levels were assessed to gain a better understanding of NF-κB signaling with IKKε inhibition (Fig. 2c). As with the siRNA against *IKBKE*, the most significantly increased proteins were the non-canonical NF-κB proteins p100/52 and RelB.

**Fig. 3:**
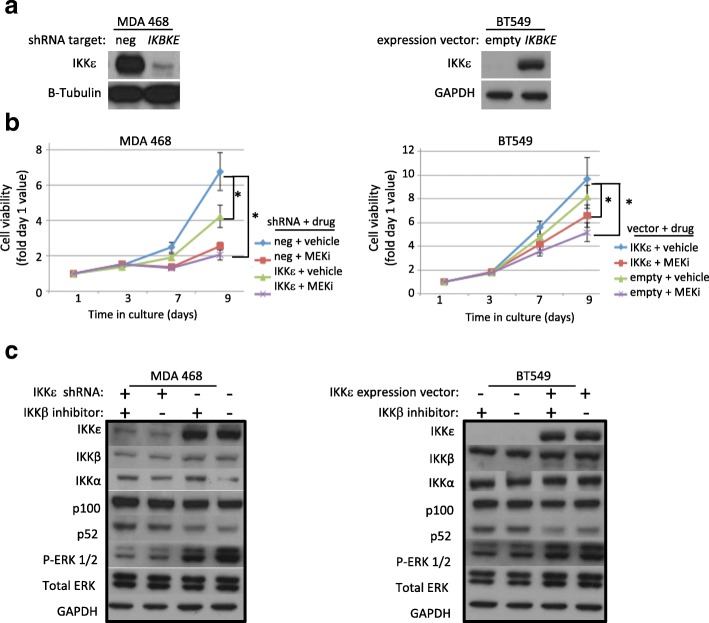
IKKε expression or activity suppresses non-canonical NF-κB protein expression. **a**) 50 μg of protein was analyzed in whole cell lysates of breast cancer cells engineered to express a constitutively active shRNA against the *IKBKE* transcript (shIKKε), left, or an expression vector for constitutive synthesis of *IKBKE*, right. Representative blots show efficiency of shIKKε and expression vector after 8 days selection in puromycin or neomycin, respectively. **b**) Left, using the MDA-MB-468 cells with stable *IKBKE* shRNA activity we measured the viability of the cells over a 9 day period. Loss of IKKε significantly impaired survival compared to control. This difference was abrogated in the presence of 1 μM MEK inhibitor. Right, the same experiment was performed using the BT549 cells stably transfected with the *IKBKE* expression vector. * day 9 significantly different from corresponding vehicle control *P* < 0.05, one-way ANOVA, post hoc Tukey. **c**) Left, western blot analysis of MDA MB 468 cells with shRNA-mediated knockdown of *IKBKE* shows decline in activated MEK (phosphorylated ERK1/2) and increase in p52 levels compared to negative control. Right, stable expression of wild type IKKε is correlated with increased activated MEK and decreased p52. Twenty-four hour exposure 2 μM IKKβ inhibitor had no effect on protein levels

To confirm specificity of ΙΚΚε function in TNBC cells *IKBKE* was knocked down in MDA MB 468 and MDA MB 231 cells and over-expressed in BT-549 (Fig. 3a). Using these complimentary systems, the role of ΙΚΚε expression and MEK activity was first assessed. As expected, loss of ΙΚΚε significantly reduced proliferation over 9 days compared to vector control cells. Addition of a MEK inhibitor further reduced viability regardless of ΙΚΚε expression (Fig. 3b). A similar trend was observed in BT549 cells engineered to express wild-type ΙΚΚε. In addition, knockdown of *IKBKE* resulted in decreased phosphorylation of ERK, and stable over-expression of ΙΚΚε caused increased ERK phosphorylation (Fig. 3c). Taken together, these results support a model where MEK activation downstream of ΙΚΚε increases the proliferation of TNBC cells. By western blot, protein levels of the IκB kinases, IKKβ and IKKα, were largely unaffected by ΙΚΚε, but p52 levels were decreased in the presence of ΙΚΚε (Fig. 3c). The increased non-canonical NF-kB protein expression with stable *IKBKE* knockdown was confirmed in the MDA 231 cells and in the MDA 468 cells using an alternate shRNA against *IKBKE* (Additional file 3: Figure S2).

**Fig. 4:**
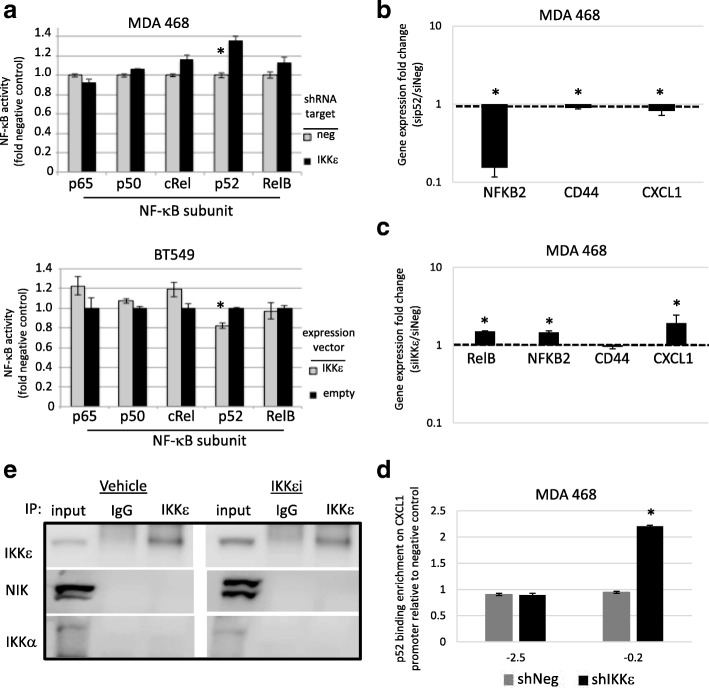
IKKε inhibits p52 activity independent of interactions with NIK and IKKα. **a**) Binding activity of the NF-κB p52 transcription factor was significantly decreased in the presence of IKKε. **b**) siRNA-mediated knockdown of *NFKB2* resulted in a significant decrease of *CD44* and *CXCL1* mRNA expression. **c**) siRNA-mediated knockdown of *IKBKE* resulted in a significant increase in mRNA expression of *RELB, NFKB2*, and *CXCL1*. **d**) CHiP-PCR experiments demonstrate significant enrichment of p52 at the promoter of *CXCL1* when IKKε is knocked down. **e**) Co-immunoprecipitation experiments indicate IKKε does not interact with NIK or IKKα. * significantly different from corresponding shNeg or siNeg control, *P* < 0.05, two-sided unpaired t-test

In order to clarify whether IKKε affected NF-κB transcription factor binding activity, an ELISA immunoassay containing a consensus NF-kB binding sequence was used to evaluate binding activity of downstream NF-κB transcription factors. Binding of the non-canonical transcription factor, p52, was significantly increased when ΙΚΚε was knocked down via shRNA relative to a non-targeting control vector (Fig. 4a, left). Similarly, in BT549 lines with endogenously low IKKε expression, binding of p52 was suppressed with exogenous expression of wild-type IKKε relative to control vector (Fig. 4a, right). This is consistent with the decreased protein level that was observed on Western blot. On the other hand, ΙΚΚε expression did not produce a difference in binding of canonical NF-κB transcription factors (p65, p50, and C-Rel). These data suggest that while ΙΚΚε supports MEK activity, it may interfere with or suppress non-canonical NF-κB activity.

Regulation of NF-κB target genes *CD44* and *CXCL1* by p52 in TNBC cells was verified using siRNA knockdown and qRT-PCR. Knockdown of *NFKB2* (the gene that encodes p100) led to a small but significant decrease in *CXCL1* expression in both lines (Fig. 4b and Additional file 4: Figure S3a) indicating transcriptional regulation of *CXCL1* by p52. *CD44* and *CXCL1* have been shown to be regulated by p52 in other systems [30]. Moreover, siRNA knockdown of *IKBKE*, which increases binding activity of p52 (Fig. 4a) led to a significant increase in *RELB, NFKB2* and *CXCL1* in the MDA 468 cells (Fig. 4c) suggesting negative transcriptional regulation of alternative NF-κB transcription factors by IKKε. In the MDA 231 cells *RELB*, *NFKB2* and *CD44* expression was increased with *IKBKE* knockdown (Additional file 4: Figure S3b). To confirm that IKKε was affecting p52 function, ChIP-PCR was performed to assess the role of IKKε on the binding of p52 in the *CXCL1* promoter. A two-fold enrichment of p52 binding occurred at a binding site 200 bases upstream of the transcription start site (Fig. 4d). A similar trend was seen in the MDA 231 cells and MDA468 cells transfected with an alternate shRNA against *IKBKE* (shIKKε 2) (Additional file 4: Figure S3c-d). These data confirm that IKKε negatively affects the transcription factor activity of p52. A co-immunoprecipitation assay was performed to determine if IKKε is binding to NF-kB inducing Kinase (NIK) or IKKα, two upstream kinases responsible for activation of the non-canonical NF-κB pathway (Fig. 4e). Neither NIK nor IKKα co-immunoprecipitated with IKKε suggesting IKKε regulates p52 activity through gene transcription and not proteosomal processing of p100.

**Fig. 5:**
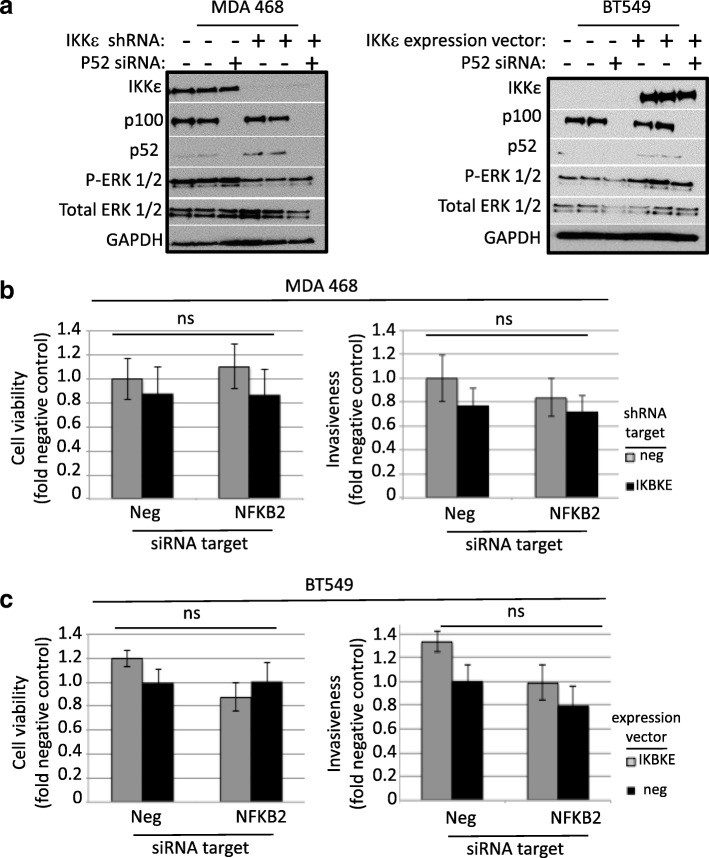
p52 does not contribute to viability or invasive potential. **a**) Using the MDA-MB-468 cells with stably knocked down *IKBKE*, we transiently knocked down *NFKB2* using siRNA interference. Representative western blots shown using 30 μg protein lysate collected 72 h following siRNA transfection. **b**) Cell viability over 72 h was not significantly altered by loss of IKKε or p52, left. Similarly, loss of IKKε or p52 had no effect on invasiveness at 72 h, right. **c**) Viability, left, and invasion, right, experiments were repeated using the BT549 cells stably expressing IKKε. ns, not significant according to one-way ANOVA, post hoc-Tukey

The role of p52 and non-canonical NF-κB activation in breast cancer cells is unclear. Since its expression is inversely correlated with ΙΚΚε, the non-canonical NF-κB pathway likely supports a function that is either not dependent on ΙΚΚε or repressed by ΙΚΚε. Indeed, the heterogeneity of TNBC cells suggests distinct mechanisms are operating in individual cells to support and maintain phenotypic variability among the population. Therefore, this study interrogated key attributes of cancer cells: proliferation, invasion, resistance to Resistance to anoikis, and spheroid formation. To further explore these aspects, *NFKB2* was transiently knocked down using siRNA interference in breast cancer cells expressing high or low levels of ΙΚΚε. Loss of p52 had no effect on the levels of ΙΚΚε or MEK, suggesting their expression or activation are not regulated by non-canonical NF-κB signaling (Fig. 5a). Furthermore, there was little or no change in viability or invasion potential over 72 h with loss of p52 (Fig. 5b-c).

**Fig. 6:**
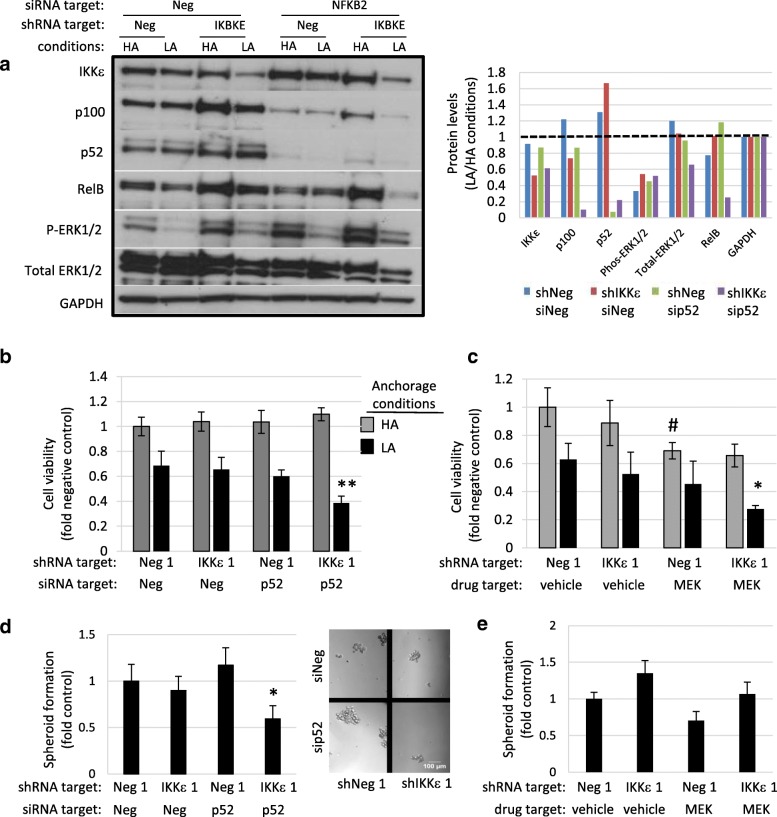
p52 supports viability and spheroid formation in anchorage resistant LA environment. **a**) Western blot and quantification demonstrating increased p52 protein levels and decreased activated MEK levels in LA relative to HA conditions. **b**) Loss of p52 or IKKε alone had no effect on viability in anchorage supportive conditions or anchorage resistant conditions after 48 h culture. Loss of both IKKε and p52 significantly reduced survival in the anchorage resistant conditions. Statistical analysis: ** indicates condition significantly different when compared to all HA and LA conditions, one-way ANOVA, post hoc-Tukey. **c**) Pharmacological inhibition of MEK significantly reduced survival in HA conditions and in the LA conditions when IKKε is also absent. Statistical analysis: * indicates condition significantly different from shNeg with vehicle in LA conditions; # indicates condition significantly different from shNeg with vehicle HA conditions, one-way ANOVA, post hoc-Tukey. **d**-**e**) Spheroid formation efficiency at 72 h under LA conditions is supported by p52, but not MEK. Statistical analysis: * indicates condition significantly different from neg shRNA, one-way ANOVA, post hoc-Tukey

Since ΙΚΚε is important for viability of TNBCs, it is important to know whether this phenomenon is also true in low attachment (LA), where cells grow as spheroids after seeding in ultra-low attachment flasks, compared with cells grown as a monolayer in standard high attachment (HA) culture conditions. Survival and growth in LA conditions implicates resistance to anoikis, apoptosis that occurs in absence of extracellular matrix, and the potential for spheroid growth, both of which are features of carcinogenic cells. Protein levels were assessed by western blot with IKKε and/or p52 knockdown in HA and LA conditions in MDA 468 cells (Fig. 6a). IKKε and phosphorylated ERK1/2 protein levels decreased in LA conditions whereas p52 levels increased. These data suggest that while ΙΚΚε and MEK support proliferation in both HA and LA conditions, p52 provides a survival advantage specifically in conditions with low anchorage support, indicative of a potential role in spheroid growth. Interestingly, when cultured in LA conditions, knockdown of either *IKBKE* or *NFKB2* alone did not affect relative viability (Fig. 6b and Additional file 5: Figure S4a). Knockdown of both *IKBKE* and *NFKB2*, however, significantly reduced viability in LA conditions. MEK inhibition decreased viability in both anchorage-resistant (LA) and anchorage–supportive (HA) plates, and the combination of MEK inhibition and *IKBKE* knockdown was the most detrimental to cell growth (Fig. 6c and Additional file 5: Fig. 4b-c). Since p52 supports unattached growth, in vitro spheroid formation was examined. Spheroid formation efficiency was analyzed in the presence and absence of ΙΚΚε and/or p52 using knockdown of *IKBKE* and *NFKB2*, respectively. Once again, loss of either ΙΚΚε or p52 alone had no effect on spheroid formation efficiency, but loss of both ΙΚΚε and p52 significantly reduced spheroid formation potential (Fig. 6d and Additional file 6: Figure S5a-b). Although MEK signaling is important for cell growth and viability, MEK inhibition did not have a significant effect on spheroid formation (Fig. 6e and Additional file 6: Figure S5c).

In summary, this work proposes a model where ΙΚΚε and MEK are important for overall proliferative capacity of TNBC cells in all growth conditions, while non-canonical NF-κB signaling through p52 is important for survival in anchorage-resistant LA environments. It may be beneficial to the cell to turn off 3D spheroid survival signals when cells are growing in a solid tumor formation attached to extracellular matrix in order to mobilize cellular resources towards replication. Under 3D unattached conditions, such as when metastasizing through the blood stream, both ΙΚΚε and p52 may be needed to maintain cell viability in suspension at the expense of high proliferation.

## Discussion

NF-κB signaling is important for cancer development, yet the functional role that each subunit plays in this process has not been fully elucidated. IKKε has diverse functions, from activating IRF3 and IRF7 as part of an anti-viral response [31]. This kinase has also been shown to activate canonical NF-kB signaling by directly phosphorylating the subunit RelA/p65 [32]. ΙΚΚε is an oncogene in breast cancer with constitutive expression in some breast cancer cell lines and patient samples. Several studies, including this one, shed light on the functional significance of this protein in breast cancer progression [33, 34]. The role of ΙΚΚε is of particular importance given the lack of therapeutic targets in TNBC and the deficient characterization of this protein.

The kinase activity of ΙΚΚε positively regulates the promoter regions of cyclin D1 and RelB in TNBC, and loss of ΙΚΚε activity diminished the cells ability to grow in soft agar and form colonies in Matrigel [33]. In another setting, ΙΚΚε controlled constitutive phosphorylation of p65 to positively regulate proliferation of HeLa cells [34]. Also in HeLa cells, ΙΚΚε directly interacts with a complex containing both p65 and p52, and phosphorylation of p65 by ΙΚΚε resulted in the transactivation of p52 [35]. In contrast to HeLa cells, our data show that ΙΚΚε increases MEK activation and decreases p52 activity in TNBC. This study further shows that protein levels of p52 increase while activated MEK decreases in LA conditions, regardless of IKKε expression. These data suggest that ΙΚΚε supports long-term viability in diverse environments but that p52 is required to maintain this property in anchorage resistant LA cultures, where cell death occurs within days. Non-canonical NF-κB signaling assists in the development of breast cancer spheroids and may play a larger role in 3-D growth dynamics compared with MEK signaling. This study expands on recent reports by our laboratory and others highlighting a role for non-canonical NF-κB signaling supporting a cancer spheroid phenotype [17, 36–38].

Together, these studies further illustrate the complex crosstalk associated with NF-κB signaling in maintaining cancer cell survival, and highlight the varied roles of ΙΚΚε. This diversity is especially critical in the context of cancer where plasticity is advantageous for cell survival. Although IKKε expression is inversely proportional to p52, both are required for growth in LA. This suggests that, while some of their functions may overlap in HA conditions, these two proteins may also serve independent roles in sustaining the LA cells. Current literature states that canonical and non-canonical substrates can be targeted by ΙΚΚε to induce NF-κB activation [39]. Importantly, both canonical and non-canonical NF-κB signals support cancer cell progression and the crosstalk with other signaling pathways provides additional mechanisms for oncogenesis [21, 37]. This may be especially important in a changing tumor cell microenvironment. For example, in the reversible process of epithelial-to-mesenchymal transition, cellular requirements differ as the cell fluxes between a solid primary tumor, shedding into the lympho-vascular system, and re-settling into a micrometastatic niche. Breast cancer stem cells are known to have this capacity to transition between epithelial and mesenchymal states, and it has been shown that NF-κB can regulate this process [37, 40]. The complex interaction between IKKε and p52 requires further study in order to identify the optimal point of intervention when designing therapeutics to target these pathways.

In light of these findings and our current study, it is plausible that ΙΚΚε supports proliferation via canonical MEK activation in anchorage supportive conditions and viability via non-canonical NF-κB p52 signaling in anchorage-resistant conditions. These findings underscore the key role of ΙΚΚε in both primary tumors and the metastatic process. Importantly, our previous work showed that ΙΚΚε was higher in metastatic ovarian cancer, and was crucial to the metastatic process in a mouse model [24]. The results presented herein underscore a similar function for ΙΚΚε in TNBC, and designate a cooperative role for non-canonical NF-κB signaling in resisting anoikis and forming spheroids.

## Conclusions

There is dire need to develop better combinatorial therapeutics for combating TNBC. ΙΚΚε is a diverse kinase capable of supporting viability of TNBC cells under different cellular contexts. Our study suggests that IKKε cooperates with MEK to support proliferation whereas IKKε directs cellular resources to p52 to support viability in an anchorage resistant or mobile setting. A better understanding of the function of IKKε within the NF-κB signaling network as well as with other major signaling pathways will likely provide alternative therapeutic strategies for patients with TNBC.

## Additional files

Additional file 1: **Table S1.** IC50 values for selected inhibitors in breast cancer lines. IC50 values obtained in the current study listed with IC50 values in same cell lines obtained from public database www.cancerrxgene.org. (PPTX 40 kb)

Additional file 2: **Figure S1.** Protein activity suppressed by inhibitors. Western blot quantification and short time point for phospho-ERK western blot. a) Quantification of western blot represented in Figure 2a showing protein level changes in BT549 or MDA MB 468 cells after 6 h treatment with indicated inhibitors. Quantification is relative to vehicle control lanes. b) Western blot showing decrease in phosphorylated ERK1/2 upon 30-min exposure to IKKε inhibitor, and accompanying graph for quantitation of phospho-ERK, as normalized to GAPDH, and relative to expression in untreated MDA468. (PPTX 429 kb)

Additional file 3: **Figure S2.** Knockdown of IKKε leads to increased expression of non-canonical NF-kB proteins in at least two TNBC lines. Western blot and quantification of additional shRNA and data in MDA MB 231 cell line. An alternate shRNA sequence against IKBKE was expressed in MDA MB 468 cells and in MDA MB 231 cells to show specificity and an additional TNBC model. (PPTX 200 kb)

Additional file 4: **Figure S3.** IKKε inhibits activity of p52. qRT-PCR and ChIP-PCR results for MDA MB 231 cell line and additional shRNA in MDA MB 468 cell line. a) siRNA-mediated knockdown of NFKB2 in MDA MB 231 cells led to a significant decrease in CXCL1 expression. b) siRNA-mediated knockdown of IKBKE in MDA MB 231 cells increased expression of RELB, NFKB2, and CD44. c) Loss of IKKε in MDA MB 231 cells led to a significant enrichment of p52 binding on the promoter of the CXCL1 gene. d) Similar results were seen in MDA MB 468 cells expressing an alternate shRNA against IKBKE (shIKKε 2). (PPTX 64 kb)

Additional file 5: **Figure S4.** IKKε and p52 or MEK supports viability in LA conditions in at least two TNBC lines. Growth conditions and anoikis data with additional shRNA in MDA MB 468 cell line and in MDA MB 231 cell line. a) Left, expressing an alternate shRNA for IKBKE in MDA MB 468 cells supports the data shown in Fig. 6b. Right, similar trends were also seen in the MDA MB 231 line. b) MEK inhibition in presence of alternate shRNA against IKBKE led to similar outcomes as shown in Figure 6c. Viability of MDA MB 231 cells is more dependent on MEK signaling than IKKε. c) Western blot verifying IKKε and p52 knockdown in MDA MB 231 cells. Statistical analysis: * indicates condition significantly different as indicated by bars; ** indicates condition significantly different when compared to all HA and LA conditions, one-way ANOVA, post hoc-Tukey. (PPTX 392 kb)

Additional file 6: **Figure S5.** IKKε and p52 or MEK supports viability in LA conditions in at least two TNBC lines. Spheroid formation data with additional shRNA in MDA MB 468 cell line and in MDA MB 231 cell line. a) An alternate shRNA for IKBKE in MDA MB 468 cells supports the data shown in Figure 6d that p52 and IKKε are both necessary for efficient spheroid formation. b) The MDA MB 231 was more dependent on p52 for efficient spheroid formation as knockdown of IKKε had no effect or slightly enhanced spheroid formation c) MEK had no effect on spheroid formation in MDA MB 231 cells however knockdown of IKKε enhanced spheroid formation efficiency. (PPTX 368 kb)

## Abbreviations

BME: Basement membrane extract
EGF: Epidermal growth factor
ERK: Extracellular signal-related kinase
FBS: Fetal bovine serum
FGF: Fibroblast growth factor
HA: High attachment culture conditions
HER2: Human epidermal growth factor receptor 2
IKKα: Inhibitor of nuclear factor kappa-B kinase subunit alpha
IKKβ: Inhibitor of nuclear factor kappa-B kinase subunit beta
IKKε: Inhibitor of nuclear factor kappa-B kinase subunit epsilon
LA: Low attachment culture conditions
MEK: Mitogen-activated protein/extracellular signal-regulated kinase kinase
NF-κB: Nuclear factor kappa-light-chain-enhancer of activated B cells
ns: Not significant
RPMI: Roswell Park Memorial Institute
shRNA: Short hairpin RNA
siRNA: Short interfering RNA
TNBC: Triple-negative breast cancer
XTT: Tetrazolium dye
