# Supplementary material for: IΚΚε cooperates with either MEK or non-canonical NF-kB driving growth of triple-negative breast cancer cells in different contexts
Source: BMC Cancer. 2018 May 25;18:595. doi: 10.1186/s12885-018-4507-2 (PMC5970439; doi:10.1186/s12885-018-4507-2)
Supplement: Supplementary file 1 — Table S1. IC50 values for selected inhibitors in breast cancer lines. IC50 values obtained in the current study listed with IC50 values in same cell lines obtained from public database www.cancerrxgene.org. (PPTX 40 kb) [file 12885_2018_4507_MOESM1_ESM.pptx]

## Slide 1
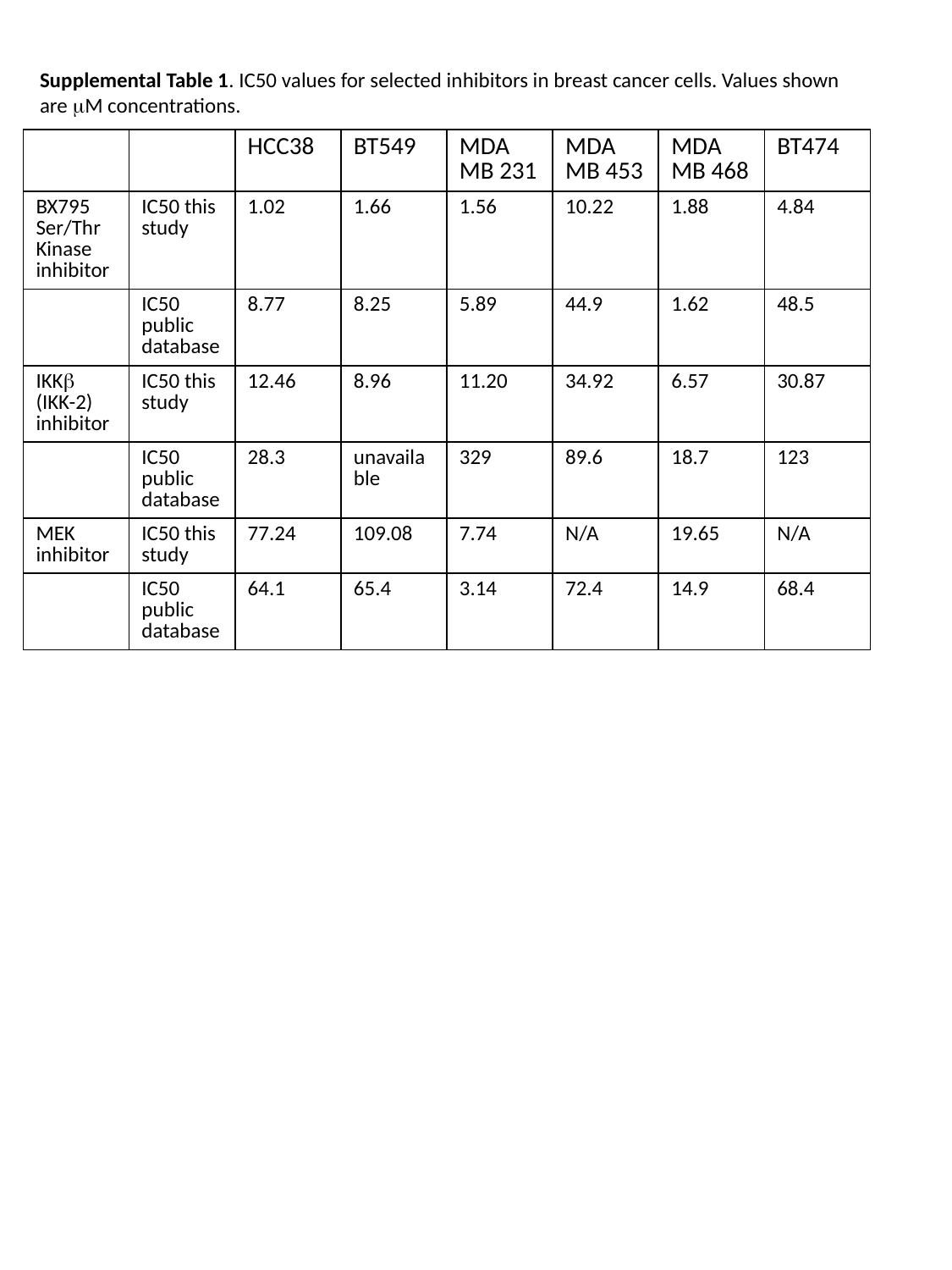

Supplemental Table 1. IC50 values for selected inhibitors in breast cancer cells. Values shown are mM concentrations.
| | | HCC38 | BT549 | MDA MB 231 | MDA MB 453 | MDA MB 468 | BT474 |
| --- | --- | --- | --- | --- | --- | --- | --- |
| BX795 Ser/Thr Kinase inhibitor | IC50 this study | 1.02 | 1.66 | 1.56 | 10.22 | 1.88 | 4.84 |
| | IC50 public database | 8.77 | 8.25 | 5.89 | 44.9 | 1.62 | 48.5 |
| IKKb (IKK-2) inhibitor | IC50 this study | 12.46 | 8.96 | 11.20 | 34.92 | 6.57 | 30.87 |
| | IC50 public database | 28.3 | unavailable | 329 | 89.6 | 18.7 | 123 |
| MEK inhibitor | IC50 this study | 77.24 | 109.08 | 7.74 | N/A | 19.65 | N/A |
| | IC50 public database | 64.1 | 65.4 | 3.14 | 72.4 | 14.9 | 68.4 |
