# Supplementary material for: IΚΚε cooperates with either MEK or non-canonical NF-kB driving growth of triple-negative breast cancer cells in different contexts
Source: BMC Cancer. 2018 May 25;18:595. doi: 10.1186/s12885-018-4507-2 (PMC5970439; doi:10.1186/s12885-018-4507-2)
Supplement: Supplementary file 2 — Figure S1. Protein activity suppressed by inhibitors. Western blot quantification and short time point for phospho-ERK western blot. a) Quantification of western blot represented in Figure 2a showing protein level changes in BT549 or MDA MB 468 cells after 6 h treatment with indicated inhibitors. Quantification is relative to vehicle control lanes. b) Western blot showing decrease in phosphorylated ERK1/2 upon 30-min exposure to IKKε inhibitor, and accompanying graph for quantitation of phospho-ERK, as normalized to GAPDH, and relative to expression in untreated MDA468. (PPTX 429 kb) [file 12885_2018_4507_MOESM2_ESM.pptx]

## Slide 1
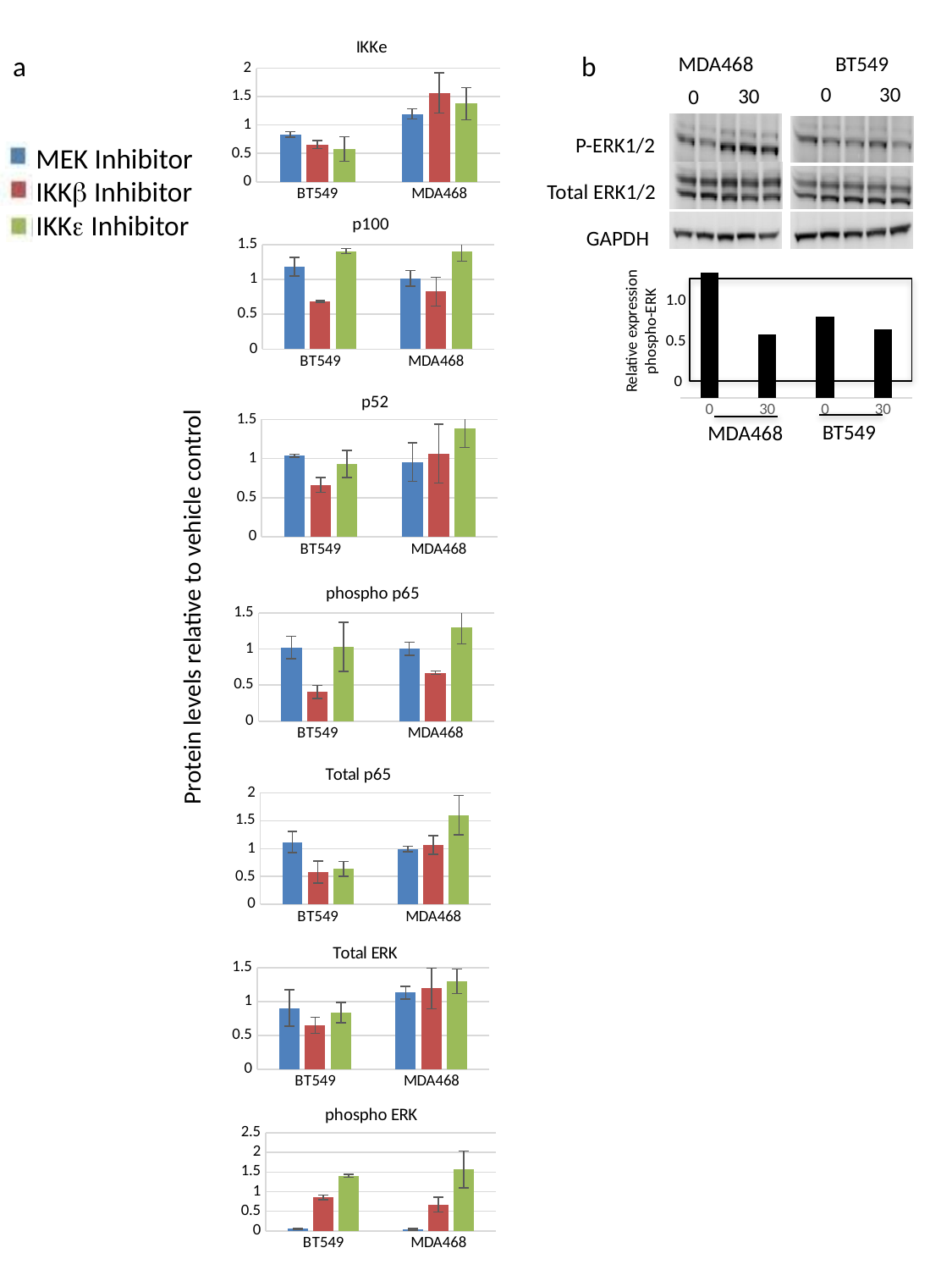

### Chart: IKKe
| Category | MEK Inhibitor | IKKB Inhibitor | IKKE Inhibitor |
|---|---|---|---|
| BT549 | 0.832071222284418 | 0.656193936923979 | 0.577777292351565 |
| MDA468 | 1.197119926628086 | 1.565734328843732 | 1.377374482670404 |a
b
MDA468
BT549
0
30
30
0
P-ERK1/2
Total ERK1/2
GAPDH
MEK Inhibitor
IKKb Inhibitor
IKKe Inhibitor
### Chart: p100
| Category | MEK Inhibitor | IKKB Inhibitor | IKKE Inhibitor |
|---|---|---|---|
| BT549 | 1.180554996858811 | 0.682850517947158 | 1.406667650286615 |
| MDA468 | 1.0136109456714 | 0.825011407133715 | 1.399248396707488 |
### Chart
| Category | relative to GAPDH |
|---|---|
| 0.0 | 0.00327862709832134 |
| 30.0 | 0.00165678409487269 |
| 0.0 | 0.00212865497076023 |
| 30.0 | 0.00178612254229538 |
1.0
Relative expression phospho-ERK
0.5
0
### Chart: p52
| Category | MEK Inhibitor | IKKB Inhibitor | IKKE Inhibitor |
|---|---|---|---|
| BT549 | 1.035516365556507 | 0.664575144438656 | 0.930970270692626 |
| MDA468 | 0.955034196352166 | 1.064673156718672 | 1.387155849097971 |BT549
MDA468
### Chart: phospho p65
| Category | MEK Inhibitor | IKKB Inhibitor | IKKE Inhibitor |
|---|---|---|---|
| BT549 | 1.020374282669094 | 0.403968762972658 | 1.030503560257667 |
| MDA468 | 1.004122324084233 | 0.671419340164496 | 1.304586637541165 |Protein levels relative to vehicle control
### Chart: Total p65
| Category | MEK Inhibitor | IKKB Inhibitor | IKKE Inhibitor |
|---|---|---|---|
| BT549 | 1.11841023627594 | 0.57881349321329 | 0.638852777123096 |
| MDA468 | 0.994978321057014 | 1.06656085951481 | 1.599188733730452 |
### Chart: Total ERK
| Category | MEK Inhibitor | IKKB Inhibitor | IKKE Inhibitor |
|---|---|---|---|
| BT549 | 0.904986849614511 | 0.646844694370095 | 0.8356075646124 |
| MDA468 | 1.13391010552986 | 1.193740022925554 | 1.297809221660324 |
### Chart: phospho ERK
| Category | MEK Inhibitor | IKKB Inhibitor | IKKE Inhibitor |
|---|---|---|---|
| BT549 | 0.052519178204674 | 0.852120087233459 | 1.410979761576543 |
| MDA468 | 0.0408916048631841 | 0.672307279547475 | 1.564603527141232 |
