# Supplementary material for: IΚΚε cooperates with either MEK or non-canonical NF-kB driving growth of triple-negative breast cancer cells in different contexts
Source: BMC Cancer. 2018 May 25;18:595. doi: 10.1186/s12885-018-4507-2 (PMC5970439; doi:10.1186/s12885-018-4507-2)
Supplement: Supplementary file 3 — Figure S2. Knockdown of IKKε leads to increased expression of non-canonical NF-kB proteins in at least two TNBC lines. Western blot and quantification of additional shRNA and data in MDA MB 231 cell line. An alternate shRNA sequence against IKBKE was expressed in MDA MB 468 cells and in MDA MB 231 cells to show specificity and an additional TNBC model. (PPTX 200 kb) [file 12885_2018_4507_MOESM3_ESM.pptx]

## Slide 1
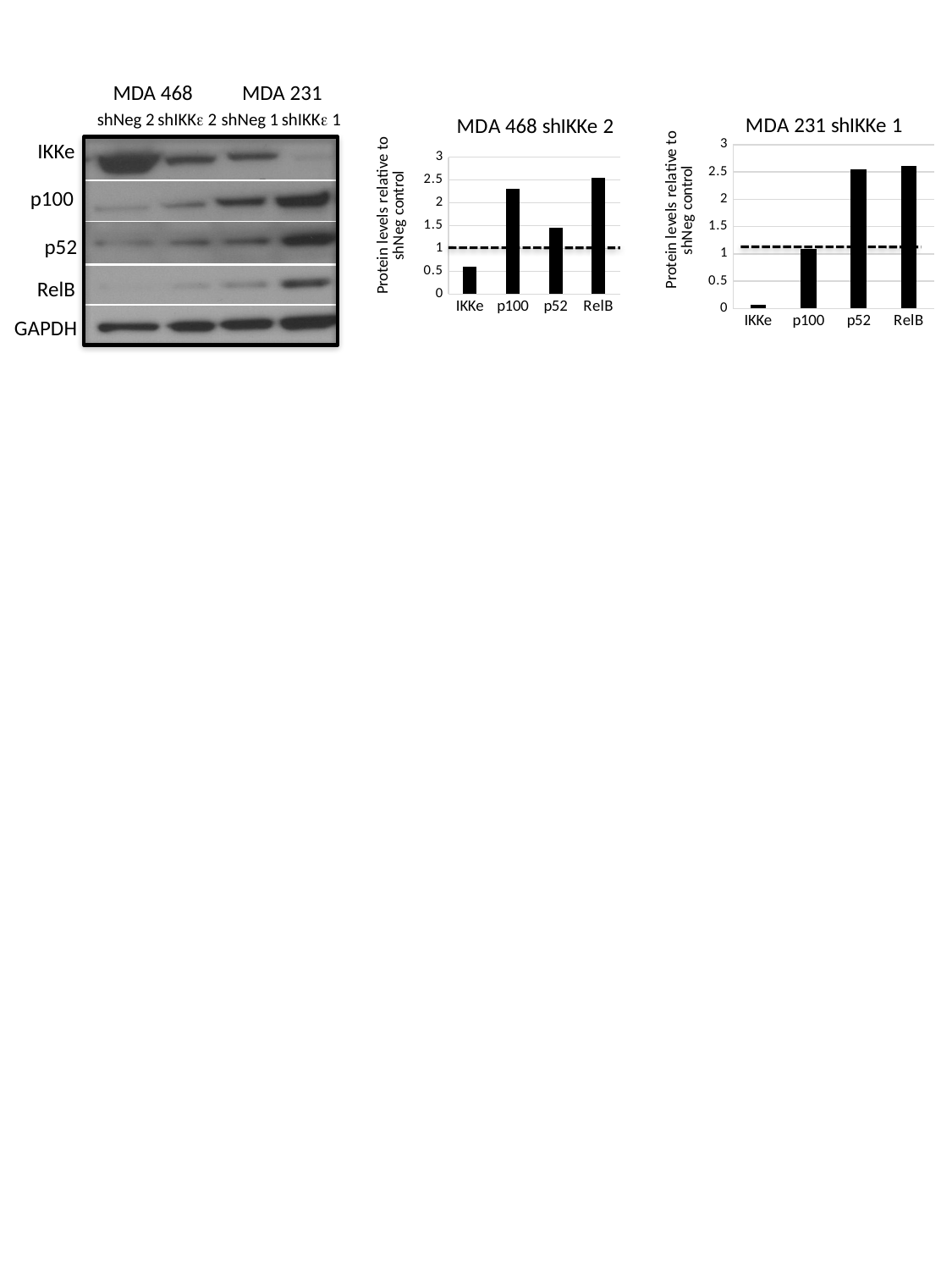

MDA 468
MDA 231
### Chart: MDA 231 shIKKe 1
| Category | MDA231 |
|---|---|
| IKKe | 0.0599387387458848 |
| p100 | 1.092226433714528 |
| p52 | 2.547301165609107 |
| RelB | 2.604799932879036 |shNeg 2
shIKKe 2
shNeg 1
shIKKe 1
### Chart: MDA 468 shIKKe 2
| Category | MDA468 |
|---|---|
| IKKe | 0.590826699207382 |
| p100 | 2.313829763381335 |
| p52 | 1.443722805294215 |
| RelB | 2.547484392300106 |IKKe
p100
p52
RelB
GAPDH
