# Supplementary material for: IΚΚε cooperates with either MEK or non-canonical NF-kB driving growth of triple-negative breast cancer cells in different contexts
Source: BMC Cancer. 2018 May 25;18:595. doi: 10.1186/s12885-018-4507-2 (PMC5970439; doi:10.1186/s12885-018-4507-2)
Supplement: Supplementary file 4 — Figure S3. IKKε inhibits activity of p52. qRT-PCR and ChIP-PCR results for MDA MB 231 cell line and additional shRNA in MDA MB 468 cell line. a) siRNA-mediated knockdown of NFKB2 in MDA MB 231 cells led to a significant decrease in CXCL1 expression. b) siRNA-mediated knockdown of IKBKE in MDA MB 231 cells increased expression of RELB, NFKB2, and CD44. c) Loss of IKKε in MDA MB 231 cells led to a significant enrichment of p52 binding on the promoter of the CXCL1 gene. d) Similar results were seen in MDA MB 468 cells expressing an alternate shRNA against IKBKE (shIKKε 2). (PPTX 64 kb) [file 12885_2018_4507_MOESM4_ESM.pptx]

## Slide 1
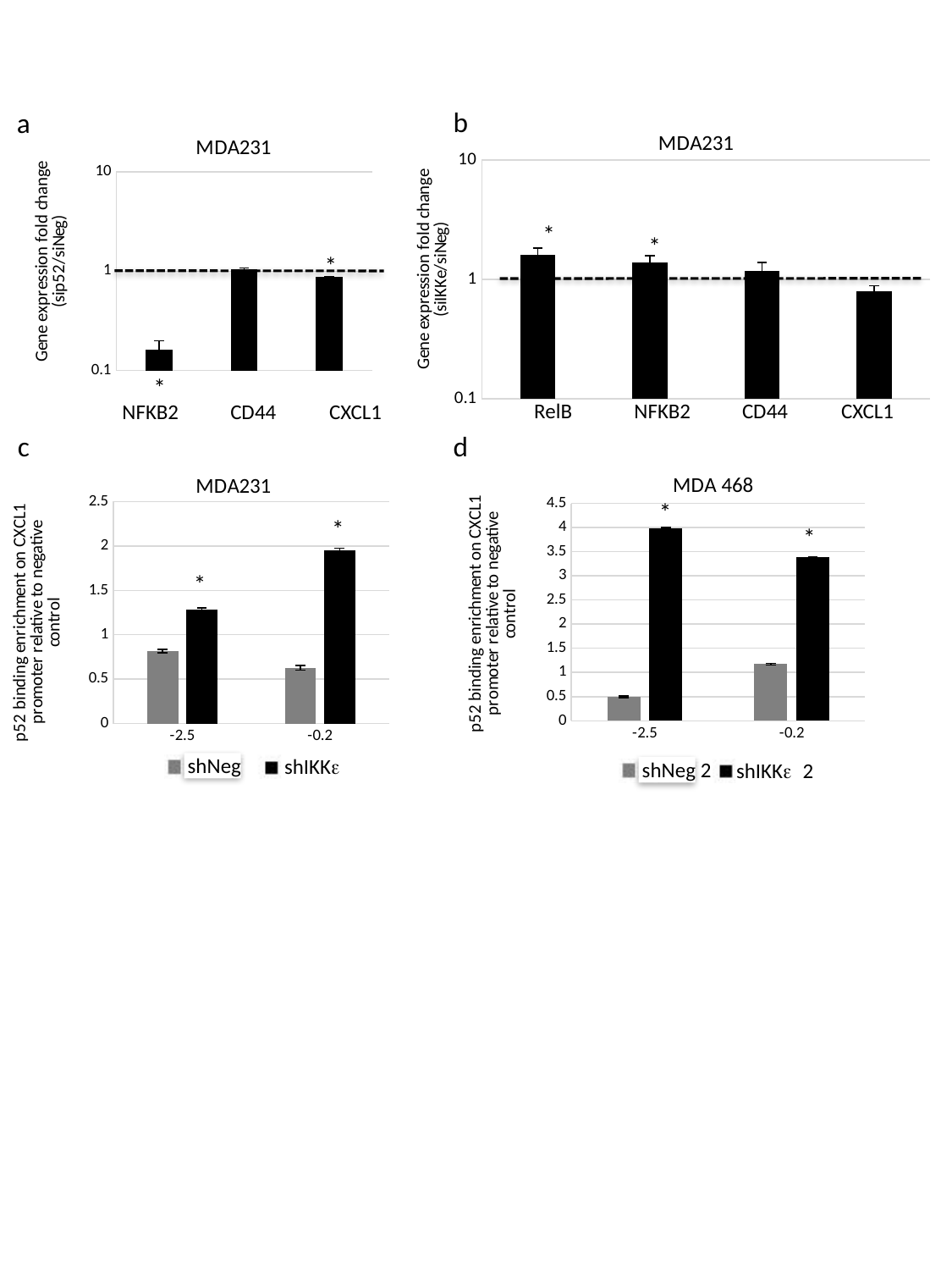

b
### Chart: MDA231
| Category | |
|---|---|
| RelB | 1.61 |
| p52 | 1.376088969060107 |
| CD44 | 1.171759081470901 |
| CXCL1 | 0.796680500069568 |a
### Chart: MDA231
| Category | |
|---|---|
| p52 | 0.161802933013571 |
| CD44 | 1.051985807207408 |
| CXCL1 | 0.88049177909537 |*
*
*
*
RelB
NFKB2
CD44
CXCL1
NFKB2
CD44
CXCL1
c
d
### Chart: MDA231
| Category | shNeg | shIKKe |
|---|---|---|
| -2.5 | 0.8175408046573 | 1.28064312631075 |
| -0.2 | 0.627949069375049 | 1.949629742894891 |
### Chart: MDA 468
| Category | shNeg | shIKKe |
|---|---|---|
| -2.5 | 0.496199152024753 | 3.979569611694191 |
| -0.2 | 1.172280636542725 | 3.3829046686384 |*
*
*
*
shNeg
shIKKe
shNeg 2
shIKKe 2
