# Supplementary material for: IΚΚε cooperates with either MEK or non-canonical NF-kB driving growth of triple-negative breast cancer cells in different contexts
Source: BMC Cancer. 2018 May 25;18:595. doi: 10.1186/s12885-018-4507-2 (PMC5970439; doi:10.1186/s12885-018-4507-2)
Supplement: Supplementary file 5 — Figure S4. IKKε and p52 or MEK supports viability in LA conditions in at least two TNBC lines. Growth conditions and anoikis data with additional shRNA in MDA MB 468 cell line and in MDA MB 231 cell line. a) Left, expressing an alternate shRNA for IKBKE in MDA MB 468 cells supports the data shown in Fig. 6b. Right, similar trends were also seen in the MDA MB 231 line. b) MEK inhibition in presence of alternate shRNA against IKBKE led to similar outcomes as shown in Figure 6c. Viability of MDA MB 231 cells is more dependent on MEK signaling than IKKε. c) Western blot verifying IKKε and p52 knockdown in MDA MB 231 cells. Statistical analysis: * indicates condition significantly different as indicated by bars; ** indicates condition significantly different when compared to all HA and LA conditions, one-way ANOVA, post hoc-Tukey. (PPTX 392 kb) [file 12885_2018_4507_MOESM5_ESM.pptx]

## Slide 1
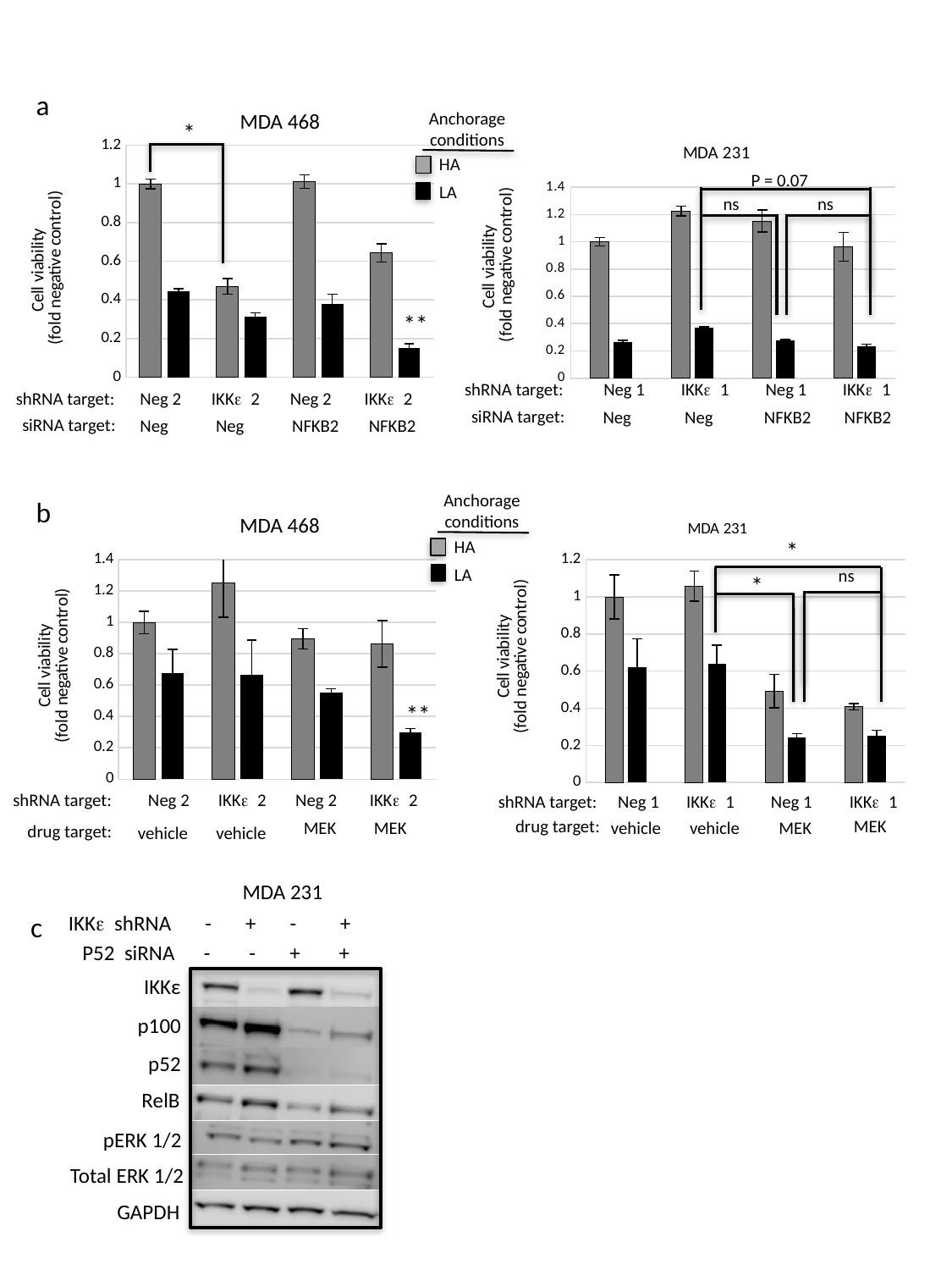

a
Anchorage
conditions
HA
LA
MDA 468
*
### Chart
| Category | HA | LA |
|---|---|---|
| shNeg siNeg | 1.0 | 0.442430026857715 |
| shIKKE siNeg | 0.468938258176167 | 0.311950243210082 |
| shNeg sip52 | 1.01215407606719 | 0.379676998337006 |
| shIKKe sip52 | 0.641989377930283 | 0.151768368808549 |
### Chart: MDA 231
| Category | HA | LA |
|---|---|---|
| shNeg siNeg | 1.0 | 0.25992855519064 |
| shIKKE siNeg | 1.225421791258682 | 0.365315405369252 |
| shNeg sip52 | 1.15203537935528 | 0.277249937684981 |
| shIKKe sip52 | 0.963249896283503 | 0.232289468432783 |P = 0.07
ns
ns
**
shRNA target:
Neg 1
IKKe 1
Neg 1
IKKe 1
siRNA target:
Neg
Neg
NFKB2
NFKB2
shRNA target:
Neg 2
IKKe 2
Neg 2
IKKe 2
siRNA target:
Neg
Neg
NFKB2
NFKB2
Anchorage
conditions
HA
LA
### Chart: MDA 231
| Category | HA | LA |
|---|---|---|
| siNeg | 1.0 | 0.621405621656601 |
| siIkke | 1.057757903473924 | 0.638076350368944 |
| siNeg+Meki | 0.492632642796839 | 0.241806472289458 |
| siIkke+ Meki | 0.407899246650726 | 0.250736349947261 |*
ns
*
shRNA target:
Neg 1
IKKe 1
Neg 1
IKKe 1
drug target:
MEK
vehicle
vehicle
MEK
b
MDA 468
### Chart
| Category | HA | LA |
|---|---|---|
| siNeg | 1.0 | 0.67761025202236 |
| siIkke | 1.252012204330196 | 0.66403059782374 |
| siNeg+Meki | 0.895034897052074 | 0.550151645339238 |
| siIkke+ Meki | 0.863310273411168 | 0.296999105809066 |**
shRNA target:
Neg 2
IKKe 2
Neg 2
IKKe 2
MEK
MEK
drug target:
vehicle
vehicle
MDA 231
c
 IKKe shRNA - + - +
 P52 siRNA - - + +
IKKε
p100
p52
RelB
pERK 1/2
Total ERK 1/2
GAPDH
