# Supplementary material for: IΚΚε cooperates with either MEK or non-canonical NF-kB driving growth of triple-negative breast cancer cells in different contexts
Source: BMC Cancer. 2018 May 25;18:595. doi: 10.1186/s12885-018-4507-2 (PMC5970439; doi:10.1186/s12885-018-4507-2)
Supplement: Supplementary file 6 — Figure S5. IKKε and p52 or MEK supports viability in LA conditions in at least two TNBC lines. Spheroid formation data with additional shRNA in MDA MB 468 cell line and in MDA MB 231 cell line. a) An alternate shRNA for IKBKE in MDA MB 468 cells supports the data shown in Figure 6d that p52 and IKKε are both necessary for efficient spheroid formation. b) The MDA MB 231 was more dependent on p52 for efficient spheroid formation as knockdown of IKKε had no effect or slightly enhanced spheroid formation c) MEK had no effect on spheroid formation in MDA MB 231 cells however knockdown of IKKε enhanced spheroid formation efficiency. (PPTX 368 kb) [file 12885_2018_4507_MOESM6_ESM.pptx]

## Slide 1
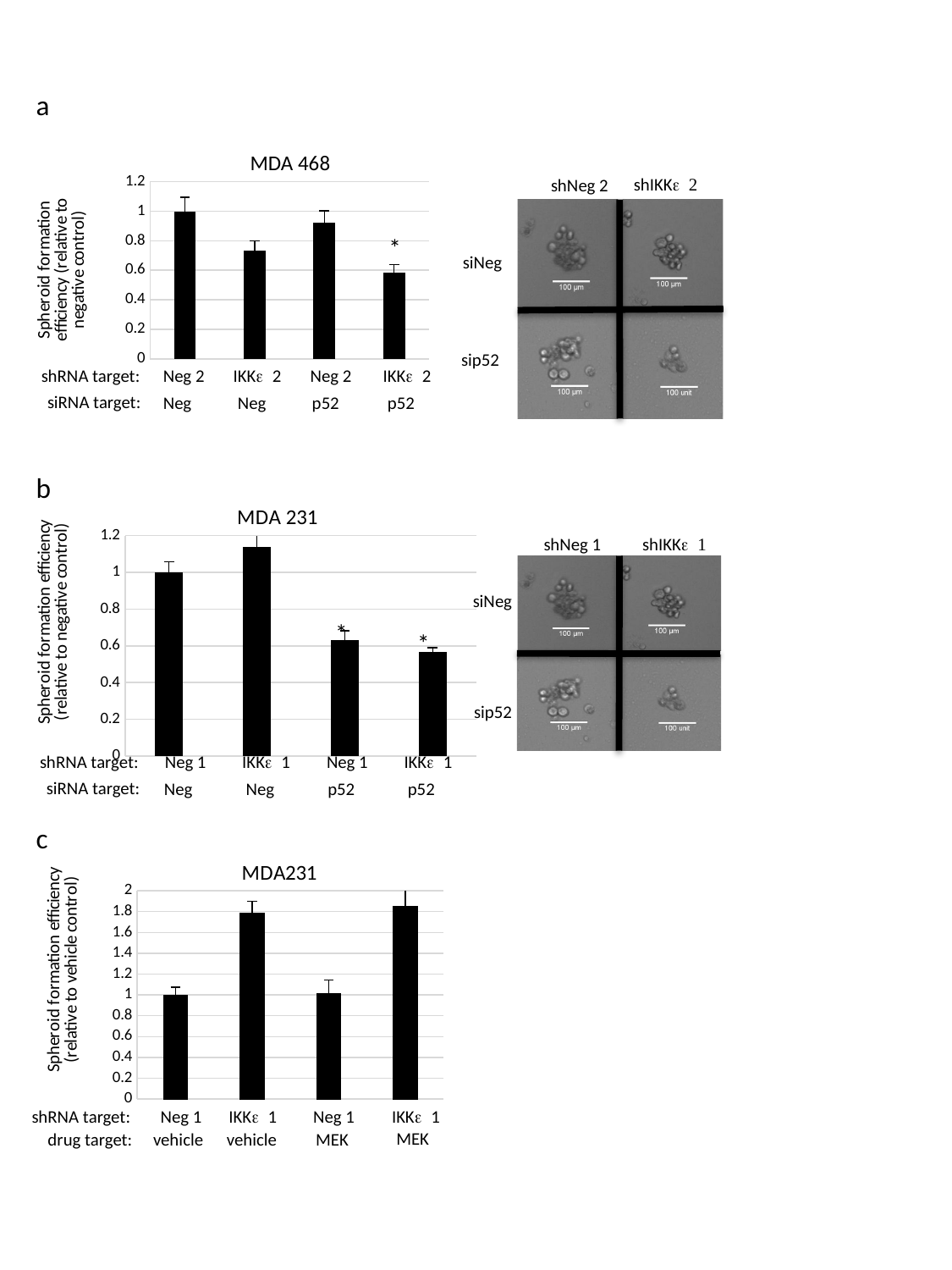

a
MDA 468
### Chart
| Category | |
|---|---|
| shNeg siNeg | 1.0 |
| shIKKe siNeg | 0.736059479553903 |
| shNeg sip52 | 0.925650557620818 |
| shIKKe sip52 | 0.58364312267658 |shIKKe 2
shNeg 2
*
siNeg
sip52
shRNA target:
Neg 2
IKKe 2
Neg 2
IKKe 2
siRNA target:
Neg
Neg
p52
p52
b
### Chart: MDA 231
| Category | |
|---|---|
| shNeg siNeg | 1.0 |
| shIKKe siNeg | 1.136363636363636 |
| shNeg sip52 | 0.630681818181818 |
| shIKKe sip52 | 0.568181818181818 |*
*
shNeg 1
shIKKe 1
siNeg
sip52
shRNA target:
Neg 1
IKKe 1
Neg 1
IKKe 1
siRNA target:
Neg
Neg
p52
p52
c
### Chart: MDA231
| Category | |
|---|---|
| shNeg vehicle | 1.0 |
| shIKKe vehicle | 1.78409090909091 |
| shNeg MEKi | 1.011363636363636 |
| shIKKe MEKi | 1.852272727272727 |*
*
shRNA target:
Neg 1
IKKe 1
Neg 1
IKKe 1
MEK
drug target:
vehicle
vehicle
MEK
